# Identification and validation of biomarkers related to oxidative stress in pediatric sepsis utilizing WGCNA and multiple machine learning algorithms

**DOI:** 10.3389/fbinf.2026.1745381

**Published:** 2026-10-12

**Authors:** Yumeng Liu, Gang Luo

**Affiliations:** Department of Pediatrics, Liaoning Maternal and Child Health Hospital, Shenyang, Liaoning, China

**Keywords:** biomarkers, machine learning, oxidative stress, pediatric sepsis, WGCNA

## Abstract

**Background:**

This research aimed to identify novel oxidative stress (OS)–related biomarkers and develop predictive models for the early detection of pediatric sepsis.

**Methods:**

Five open-access datasets from the gene expression omnibus database were analyzed, which included peripheral whole blood samples from children with sepsis and healthy controls. Using bioinformatics approaches, we identified differentially expressed genes (DEGs) associated with OS. We then combined weighted gene co-expression network analysis (WGCNA) and multiple machine learning algorithms (ML) to identify seven candidate genes, constructed a protein–protein interaction (PPI) network to screen six hub genes, and evaluated their correlations with the immune microenvironment. We subsequently evaluated the diagnostic performance of the hub genes using training datasets (GSE26440 and GSE26378) and validation datasets (GSE13904, GSE8121, and GSE9692). Moreover, molecular docking analysis revealed interactions between hub gene–encoded proteins and small-molecule compounds, which helps predict drug sensitivity.

**Results:**

The study identified 36 differentially expressed oxidative stress-related genes (OSRGs). Gene Ontology (GO) and Kyoto Encyclopedia of Genes and Genomes (KEGG) enrichment analyses highlighted these genes’ roles in regulating inflammatory responses and reactive oxygen species metabolism. Seven candidate genes were identified using machine learning algorithms. Subsequently, through protein-protein interaction (PPI) network analysis, six hub genes, namely, GADD45A, HP, MAPK14, PGD, RETN, and S100A9, were identified as having significant diagnostic potential. Immune cell deconvolution analysis of peripheral blood transcriptomes suggested that hub genes related to oxidative stress may be linked to alterations in the composition of peripheral blood immune cells. The diagnostic performance of the six hub genes was validated with area under the curve (AUC) values between 0.908 and 0.982, showing their promising diagnostic performance. Additionally, molecular docking analysis suggested that small molecules Doxycycline, Ralimetinib mesylate, Tasquinimod might interact with the identified hub genes, which opens up new treatment options.

**Conclusion:**

Our research not only reveals potential diagnostic biomarkers of pediatric sepsis but also sets the stage for future studies. These studies aim to translate our findings into clinical practice, enabling earlier and more accurate diagnoses as well as targeted therapies to improve outcomes for children.

## 1 Background

Sepsis continues to be one of the primary causes of death in children (Tan et al., 2019). The condition results from a dysregulated immune response to infection, which causes severe organ impairment (Schlapbach et al., 2024). Between 1990 and 2017, sepsis constituted nearly 20% of annual global deaths, equivalent to more than 20 lives lost every minute (Kempker and Martin, 2020). Mortality rates are significantly higher in children compared to adults. Septic shock, a severe subtype of sepsis, is marked by circulatory collapse combined with cellular and metabolic abnormalities (Singer et al., 2016; Weiss et al., 2020). This condition carries an especially high risk of death; in fact, nearly half of children diagnosed with sepsis succumb within 48 h of onset (Schlapbach et al., 2017). Therefore, rapid identification and early intervention within the critical initial hours are crucial for improving survival outcomes (De Backer et al., 2024). Pediatric sepsis is characterized by an abrupt onset, rapid progression, and considerable clinical heterogeneity. Early recognition is essential for improving patient outcomes. Recent international consensus guidelines emphasize that the diagnosis of sepsis in children should focus on life-threatening organ dysfunction in the setting of infection. This approach further highlights the need for a more standardized and objective clinical and biological framework for risk assessment (Schlapbach et al., 2024). Childhood sepsis cannot be regarded as a mere miniature version of adult sepsis. Children undergo continuous development of their immune systems. The inflammatory response, alterations in organ function, and clinical diagnostic criteria following infection exhibit age-related characteristics. Consequently, candidate markers identified in adult cohorts cannot be directly applied to the diagnostic context observed in children. Therefore, screening and validating molecular markers within child cohorts can provide diagnostic insights that are more aligned with the clinical and biological characteristics of pediatric sepsis.

When reactive oxygen species (ROS) are produced in amounts greater than the body can neutralize with antioxidants, oxidative stress (OS) arises (Galley, 2011). This imbalance drives mitochondrial dysfunction, which has been identified as a central pathophysiological mechanism underlying organ failure in sepsis, both in experimental models and in clinical patients (Brealey et al., 2024; Crouser, 2004). In a study of 28 severe sepsis patients during the first 24 h of admission, Brealey D. and colleagues observed that mitochondrial dysfunction, along with antioxidant depletion and impaired energy metabolism, were key drivers of multiple organ failure. Oxidative stress is widely recognized as a key mechanism contributing to inflammatory amplification, mitochondrial dysfunction, and tissue injury in sepsis. Recent clinical studies in pediatric populations have demonstrated that affected children exhibit significant alterations in oxidative stress markers and antioxidant capacity. These findings indicate that disruption of the oxidative–antioxidant balance may play a critical role in the pathophysiology of pediatric sepsis (Arı et al., 2025). Previous research has consistently linked OS with sepsis, showing that it exacerbates inflammation and accelerates organ injury. Our findings reinforce these associations and emphasize the therapeutic potential of targeting OS pathways. Notably, OS–related genes (OSRGs) such as GADD45A, HP, MAPK14, PGD, RETN, and S100A9 emerged as critical biomarkers for pediatric sepsis, underscoring the novelty and significance of our study.

Excessive generation of ROS triggers the MAPK and PI3K/Akt signaling pathways, elevates NF-κB levels, and consequently induces the expression of pro-inflammatory genes (Behl et al., 2022; Racz et al., 2010; Kwon et al., 2003). Studies have further demonstrated that, compared to conventional inflammatory markers, elevated NF-κB levels in sepsis patients serve as stronger predictors of disease severity. These findings highlight that the inflammatory response driven by OS is primarily mediated through the activation of pro-inflammatory signaling pathways (Arnalich et al., 2000; Paterson et al., 2000). To validate this hypothesis, the present study aims to elucidate the role of OS in sepsis, evaluate its potential as a therapeutic target, and contribute to the creation of more efficient diagnostic and therapeutic strategies.

In recent years, significant progress in biomedical research has been driven by the integration of large-scale computational models with multi-omics analyses. For example, Lan et al. provide a systematic overview of the applications and challenges of large language models (LLMs) in biomedical data analysis, while also proposing innovative strategies for the advanced interpretation of high-dimensional omics data (Lan et al., 2025). At the single-cell level, a deep matrix factorization model developed by Lan et al. markedly improves clustering performance and feature extraction in scRNA-seq datasets (Lan et al., 2024a). Building on this work, subsequent studies have introduced an interpretable multi-task learning framework, scMoMtF, which supports dimensionality reduction, classification, and batch effect correction in multimodal single-cell data, thereby exemplifying integrated analysis of complex biological systems (Lan et al., 2024b). Furthermore, the DeepKEGG framework, which leverages multi-omics integration, highlights the interpretability of deep learning approaches in predicting cancer recurrence and identifying potential biomarkers (Lan et al., 2024c). Collectively, these studies provide a valuable foundation for the integration of multi-omics data and the identification of key genes in pediatric sepsis.

In contrast to existing studies on multi-omics integration and interpretable models, such as DeepKEGG and scMoMtF, the present study emphasizes the integration of weighted gene co-expression network analysis (WGCNA) with multi-algorithm feature selection. In particular, it focuses on elucidating the role of oxidative stress–related pathways in pediatric sepsis. Furthermore, by incorporating insights from prior work on in-depth single-cell data decomposition and the application of large language models in biomedical analysis, this study expands the conceptual framework for uncovering disease mechanisms driven by high-dimensional data. The growing availability of public transcriptomic datasets, combined with advances in machine learning, has established integrated analytical pipelines, encompassing differential expression analysis, co-expression network construction, specialized machine learning models, and immune cell composition profiling, as a robust strategy for biomarker discovery in pediatric sepsis. Previous studies have demonstrated that integrative approaches combining bioinformatics and machine learning can effectively identify diagnostic biomarkers for pediatric sepsis and further clarify their relationships with immune cell infiltration patterns (Xu et al., 2023). Building on this foundation, focusing specifically on oxidative stress–associated genes may enhance the biological relevance and interpretability of candidate biomarker selection. Overall, this study proposes a novel and systematic framework for integrating diverse analytical strategies within a clinically relevant context.

## 2 Methods

### 2.1 Data sources

Five transcriptomic datasets (GSE26440, GSE26378, GSE13904, GSE9692, and GSE8121) related to pediatric sepsis were obtained from the Gene Expression Omnibus (GEO) database. Data retrieval was performed using the GEOquery package (v2.70.0) in the R environment (v4.3.3). All selected datasets comprised peripheral whole-blood transcriptomic profiles from pediatric sepsis patients and were generated using the GPL570 microarray platform, ensuring a high degree of comparability in terms of sample type, disease context, and detection methodology. To reduce bias associated with individual cohorts while increasing statistical power, GSE26440 and GSE26378 were merged to form the training cohort, which was used for differential expression analysis, weighted gene co-expression network analysis (WGCNA), and candidate gene identification. In contrast, GSE13904, GSE9692, and GSE8121 were combined into an independent validation cohort to assess the robustness of the identified candidate genes and diagnostic models. Detailed information regarding sample composition, grouping, and platform characteristics is provided in Supplementary Table S1. The Series Matrix files for each dataset were retrieved using the “getGEO()” function, enabling extraction of both gene expression matrices and corresponding clinical metadata. Platform annotation files (GPL570) were downloaded to map probe identifiers to gene symbols. Annotation preprocessing involved removing probes lacking gene symbol assignments, as well as those mapped to multiple genes (indicated by the “///” delimiter), thereby ensuring a one-to-one correspondence between probes and genes. Subsequently, the “avereps()” function from the limma package (v3.58.1) was applied to calculate the average expression values for genes represented by multiple probes, resulting in a gene-level expression matrix. The processed matrix was then screened for missing values, and any rows containing missing data were excluded to maintain data quality. Finally, the expression data were standardized and transformed using log_2_ (expression level + 1) to normalize the distribution and satisfy the assumptions required for downstream differential expression analyses. All preprocessing and statistical analyses were conducted in R, with the tidyverse suite of packages (v2.0.0) employed for data manipulation and organization. In addition, the analysis integrated 817 OS–associated genes identified from previous studies (Xu et al., 2023).

### 2.2 Identification of DEGs in pediatric sepsis

To integrate expression profiles from two independent datasets (GSE26440 and GSE26378) in the training cohort and three datasets (GSE13904, GSE9692, and GSE8121) in the validation cohort, initial normalization was performed using the normalizeBetweenArrays() function. Subsequently, batch effects were corrected using the empirical Bayes method implemented in the ComBat function from the sva package (v3.48.0), incorporating phenotypic information as covariates (Leek et al., 2012). Prior to integration, each dataset underwent standardized preprocessing, including probe annotation, gene symbol mapping, merging of duplicate probes, removal of missing values, and log_2_ transformation. Although all datasets were generated using the GPL570 platform, potential variability arising from differences in sample collection times, experimental conditions, and study designs was carefully addressed. Expression distributions were assessed both before and after integration to evaluate data consistency. To avoid information leakage, the training and validation cohorts were processed and integrated independently. For the merged expression matrices, normalizeBetweenArrays was applied to standardize data distributions, followed by batch effect correction using the ComBat method from the sva package. Importantly, group labels were retained as biological variables throughout the correction process to preserve disease-related signals. The effectiveness of batch correction was assessed using principal component analysis (PCA) and expression distribution plots, ensuring that non-biological variability was minimized while maintaining meaningful differences between sepsis and control samples. Differential expression analysis was conducted using the limma package (v3.58.1), with the Benjamini-Hochberg (BH) method applied to control the false discovery rate (FDR). Differentially expressed genes (DEGs) were identified based on the following criteria: |log_2_FC| > 0.585 and an adjusted p-value (adj.P.Val) < 0.05. To visualize the results, heatmaps were generated with the “Pheatmap” R package, while volcano plots were constructed using the “ggplot2” R package. Finally, Venn diagram analysis was executed to investigate the overlap between DEGs and OS–associated genes, yielding the final set of OS–related DEGs.

### 2.3 Functional enrichment analysis

In this research, the “clusterProfiler” package in R was utilized to execute GO and KEGG enrichment analyses, applying significance thresholds of p < 0.05 and adjusted p < 0.05 (Yu et al., 2012). GO analysis was carried out to explore the functional roles of genes across various biological processes (BP), while KEGG analysis explored molecular networks and signaling pathways, thereby providing a comprehensive understanding of differential gene functions associated with pediatric sepsis. All enrichment outcomes were illustrated with the “ggplot2” package in R and presented through graphical formats such as circular plots and bubble diagrams.

In addition, a gene set enrichment analysis (GSEA) was carried out based on phenotypic characteristics. The Z-score algorithm within the “GSVA” package was applied to generate phenotypic scores, which were subsequently displayed using boxplots to assess variations in expression between sepsis patients and healthy controls (Hänzelmann et al., 2013). Moreover, GSEA stratified the sepsis samples into high- and low-expression groups in line with the median phenotypic score. This stratification enabled the investigation of associations between OS–related DEGs and transcriptional profiles in sepsis, thereby shedding light on their potential functional significance.

### 2.4 Construction of weighted gene co-expression network analysis (WGCNA)

No additional standardization, such as z-score normalization, was applied prior to network construction. The cleaned, batch-corrected log_2_ expression matrix was used as the direct input for WGCNA. To retain genes with meaningful biological variability while minimizing noise, the variance of each gene across all samples was calculated, and only those within the top 15% of variance were selected for further analysis. The goodSamplesGenes function from the WGCNA package was then applied to identify and exclude genes and samples with missing values or zero variance, thereby ensuring data quality (Langfelder and Horvath, 2008). Before network construction, hierarchical clustering of samples was performed to generate a dendrogram, allowing visualization of sample similarity and detection of potential outliers. The pickSoftThreshold function in the WGCNA package was used to evaluate the scale-free topology fit index (R^2^) and mean connectivity across a range of soft-thresholding powers (1–20). Based on these results, an appropriate soft-thresholding power (β) was selected to approximate a scale-free network topology. Using the chosen β value, an adjacency matrix was constructed and subsequently transformed into a topological overlap matrix (TOM) to measure network interconnectedness. Hierarchical clustering was then conducted based on TOM dissimilarity (1 − TOM). Gene modules were identified using the dynamic tree-cutting algorithm, with the minimum module size set to 50 genes. Module eigengenes were calculated, and pairwise correlations among them were assessed. A merging threshold of 0.4 was applied, and modules exhibiting highly similar expression profiles were merged accordingly. Finally, the correlation between clinical traits and each module was assessed using Spearman correlation analysis, allowing the identification of key sepsis-related modules for further investigation.

### 2.5 Identification of candidate genes by multiple ML

To prevent information leakage, all steps of machine learning feature screening are confined to the training dataset. The validation dataset is not used for candidate gene screening, variable importance ranking, or parameter optimization. Instead, it is solely utilized for independent performance evaluation once the final model has been established. To identify phenotype-associated genes for the diagnosis of pediatric sepsis, 6 ML algorithms, Bayesian analysis, bagged trees, LASSO, random forest (RF), learning vector quantization (LVQ), and Boruta, were applied to the OS–related DEGs (OSDEGs) derived from the training dataset. Each algorithm first identifies candidate genes based on its own preset criteria, and then retains only those candidate genes that appear in the results of all six algorithms—a threshold requiring a gene to be present in every algorithm. This rule does not rely on the sorting scores of different algorithms, and consequently does not involve tie-breaking. Ultimately, candidate genes were identified based on consistency across multiple algorithms, as illustrated using an UpSet plot. Only genes detected by all six methods (threshold ≥ 6/6) were retained as final candidates.

The data for this study consist of a publicly available bulk microarray dataset with a limited sample size. The primary objective is to perform interpretable biomarker screening and independent validation. To achieve this, traditional machine learning algorithms enhance the robustness and repeatability of the biomarker screening process by employing cross-validation techniques to handle noise and overfitting. To improve the reproducibility of the feature selection process, this study further elucidated the quantitative screening criteria for each algorithm. Specifically, LASSO regression determines the regularization parameters via cross-validation and retains genes with non-zero regression coefficients as candidate features. Bagged trees, random forests, and Naive Bayes were implemented within the recursive feature elimination framework. The classification performance of various variable combinations was assessed through cross-validation, leading to the identification of the optimal feature subset. Additionally, the Boruta algorithm computes Z-scores based on the importance of random forest variables, compares original variables with shadow features, and retains those variables classified as “Confirmed” or exhibiting stable contributions following “Tentative Rough Fix” processing. Learning vector quantization (LVQ) selects parameter combinations via 10-fold cross-validation, and evaluates candidate genes based on their variable importance scores. Genes with an importance score exceeding 0.5 are retained (Supplementary Table S2). Ultimately, only the genes identified as significant across all six machine learning methods were retained as robust candidate features. The final candidate genes are identified based on the consistent results of multiple algorithms, rather than relying solely on the predicted scores from a single algorithm.

### 2.6 Construction of an early prediction model for pediatric sepsis

An early diagnostic model for pediatric sepsis was constructed using 7 ML algorithms: Cancerclass, LogitBoost, AdaBoost, Random Forest (RF), support vector machine (SVM), k-nearest neighbors (k-NN), and Naive Bayes (NB). These algorithms were applied to the integrated training cohort, which consisted of candidate genes identified through prior screening. To optimize performance, multiple parameter settings were tested, and a 5-fold cross-validation process, carried out ten times with random seeds, was implemented for hyperparameter tuning. The CancerClass algorithm, requiring no parameter tuning, was directly trained on the integrated training cohort. The predictive performance of the constructed classifiers was subsequently evaluated, and the area under the receiver operating characteristic curve (AUC) was calculated using the integrated validation cohort (GSE13904, GSE8121, and GSE9692). Once the candidate genes are identified and the model parameters are chosen, the final classification model is employed on the integrated validation cohort. The validation set remains independent throughout the phases of feature selection, hyperparameter optimization, and final model selection. Consequently, its sole objective is to assess the diagnostic performance of the model on samples that were not included in the training process, by computing the receiver operating characteristic curve and the area under the curve. The classifier demonstrating the highest performance in the validation cohort was ultimately selected as the final predictive model for pediatric sepsis diagnosis.

### 2.7 Construction of competitive endogenous RNA (ceRNA) and protein–protein interaction (PPI) networks

We established a PPI network for OSRGs using the STRING database. Through this database, interacting genes were identified and designated as hub genes for the diagnosis of pediatric sepsis (Szklarczyk et al., 2025). Using a consistent machine learning approach, we identified seven candidate genes. Based on the constructed protein-protein interaction (PPI) network, DDAH2 was excluded due to its low connectivity. Ultimately, six hub genes were retained for further verification.

MicroRNAs (miRNAs) control gene expression post-transcription, with individual miRNAs capable of targeting multiple genes, while a single gene may be regulated by several miRNAs. To investigate these interactions, the StarBase v2.0 database (Li et al., 2014) (https://starbase.sysu.edu.cn/) was employed to predict potential mRNA–miRNA interactions associated with hub genes. Subsequently, miRNA–long non-coding RNA (lncRNA) predictions were conducted for the identified miRNAs, and relevant miRNA–lncRNA pairs were refined. To investigate the potential post-transcriptional regulatory cues of hub oxidative stress-related genes, this study developed an mRNA-miRNA-lncRNA regulatory network based on predictions from existing databases. This analysis centers on the identified hub genes, subsequently searching for miRNAs that may interact with the corresponding mRNAs and their upstream lncRNAs. The candidate regulatory relationships are visualized using Cytoscape. It is important to note that ceRNA analysis serves solely to propose potential regulatory hypotheses and should not be utilized as a foundation for diagnostic biomarker screening or model development.

### 2.8 Analyzing immune cell composition from peripheral blood transcriptomes

In this study, CIBERSORT was employed to conduct a deconvolution analysis of immune cells using transcriptome data from peripheral whole blood (Steen et al., 2020). This analysis aimed to estimate the relative proportions of 22 immune cell types in both the sepsis and control groups. The findings reflected alterations in the composition of immune cells within peripheral blood samples, rather than indicating immune cell localization at the tissue level. Stacked plots were utilized to present the relative proportions of each immune cell subpopulation. Differences between the groups were assessed using the Wilcoxon rank sum test, and the interactions among hub oxidative stress-related genes were analyzed further. To further sightsee the association between OS biomarkers and immune cells, Spearman correlation analysis was performed, with significant correlations illustrated through lollipop charts. In addition, correlation heatmaps were generated to characterize the interactions among different immune cell subsets.

### 2.9 Validation hub genes and construction a nomogram

As a first step, the expression profiles of hub genes were derived from the training dataset, and differences between the sepsis and control cohorts were analyzed using the Wilcoxon rank-sum test, with violin plots produced to visually depict the differential expression of these hub genes. To further validate the findings, differential expression analyses were performed on the validation dataset (GSE13904, GSE8121, and GSE9692). Receiver operating characteristic (ROC) curves were then generated with the R package “pROC” to evaluate the diagnostic performance of each gene by calculating the area under the curve (AUC), optimal cut-off values, 95% confidence intervals (Robin et al., 2011). Finally, a nomogram based on the hub genes was constructed using the R package “rms,” and its predictive accuracy was assessed through calibration curve analysis.

### 2.10 Molecular docking analysis of hub OSRGs with compounds

Candidate compound screening was conducted using hub oxidative stress-related genes as input. The Drug-Gene Interaction database (DGIdb) (Freshour et al., 2021) (https://dgidb.org/) was utilized to retrieve documented gene-drug associations, while the Connectivity Map (cMAP) database (https://clue.io/) were queried to identify small molecules capable of downregulating the expression of hub OSRGs. Following the initial screening via these databases, candidate compounds and their corresponding target proteins were subjected to blind molecular docking using CB-Dock2 (Eberhardt et al., 2021) (https://cadd.labshare.cn/cb-dock2/php/index.php). The binding potential was assessed based on the AutoDock Vina score. Combinations of protein and compound with a Vina score lower than −5 kcal/mol were considered to exhibit strong binding potential and were included in the final summary of candidate compounds.

## 3 Results

### 3.1 Differentially expressed OSRGs identification

The overall workflow of the data analysis is summarized in the flowchart presented in Figure 1. To explore OSRG expression changes, five publicly available datasets from the GEO repository were analyzed. To evaluate the appropriateness of dataset integration, principal component analysis (PCA) was performed on the training cohort before and after batch correction. In the PCA plots, each point represents a sample, with coordinates defined by the first two principal components (PC1 and PC2). The percentage of variance explained by each component is indicated in parentheses, and colors distinguish between datasets or batches (GSE26440 and GSE26378). Prior to batch correction, samples from the two cohorts exhibited partial overlap in the PC1–PC2 space, suggesting that baseline batch effects were relatively mild (Figure 2A; Supplementary Figure S1). Nonetheless, a discernible separation between sepsis and control samples remained evident, indicating the preservation of disease-related biological signals. Following standardization and ComBat-based batch correction, inter-dataset variability was further reduced, resulting in a more homogeneous overall distribution of samples (Figure 2B; Supplementary Figure S2). This outcome suggests that batch-associated technical variation was effectively mitigated to a considerable extent. Subsequent differential expression analysis, WGCNA, and machine learning-based feature selection were performed on this integrated expression matrix, thereby improving the robustness and stability of candidate gene identification. The validation cohort was processed using the same preprocessing pipeline but was used exclusively for independent validation of candidate genes and model performance, without participation in feature selection or hyperparameter optimization (Supplementary Figures S3–S6). Differential expression analysis was subsequently performed with the “Limma” package, applying thresholds of |log2(Fold Change)| > 0.585 and adjusted P value < 0.05, which yielded 396 significant DEGs, including 290 upregulated and 106 downregulated genes. Visualization of these results was achieved through a volcano plot (Figure 2C) and a hierarchical clustering heatmap (Figure 2D), the latter clearly distinguishing sepsis samples from controls and thereby supporting the robustness of the differential expression analysis.

**FIGURE 1:**
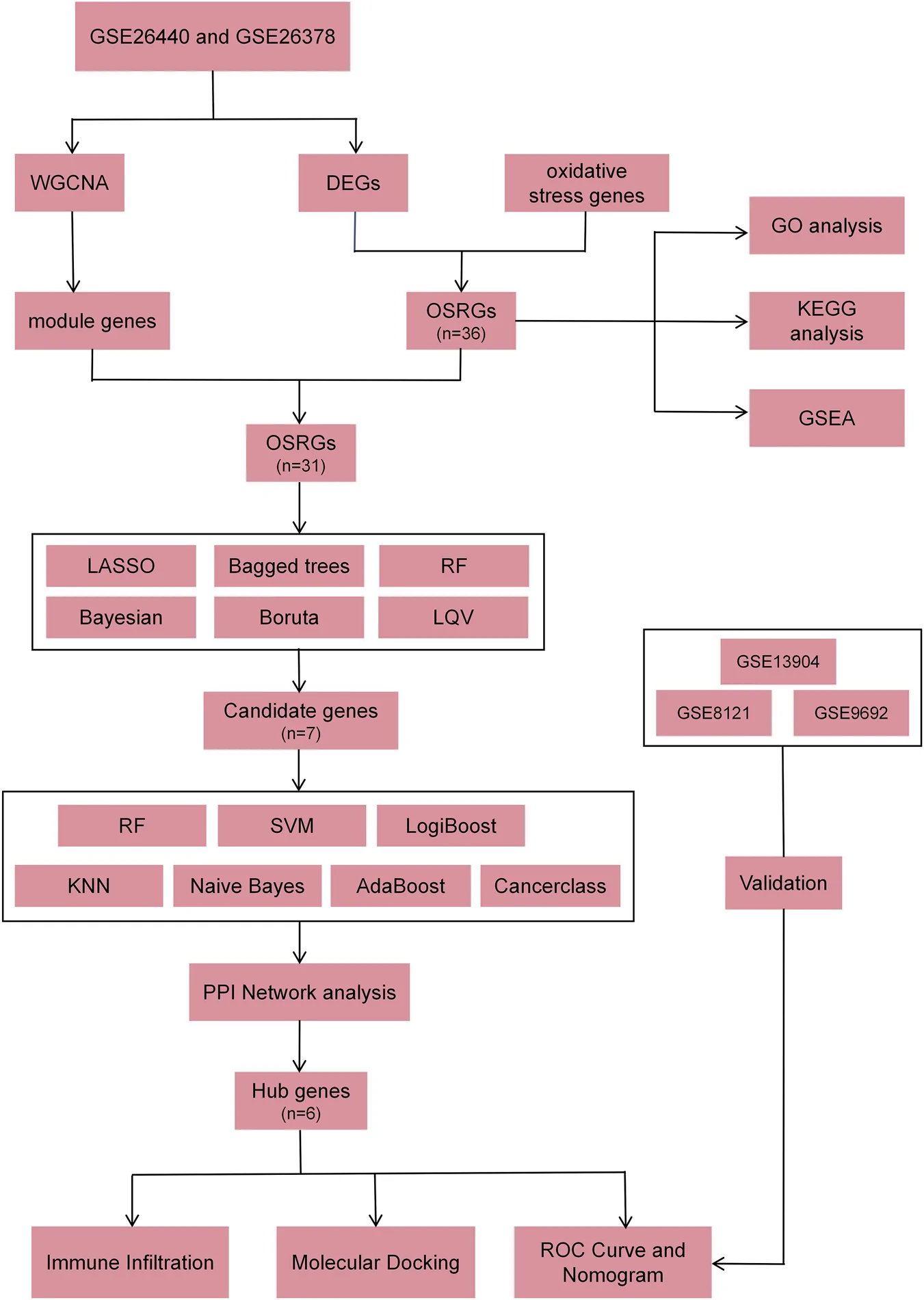
The overall flowchart of this study. Training cohort was used for feature selection, model construction, and hyperparameter tuning, whereas validation cohort was used only for independent performance evaluation.

**FIGURE 2:**
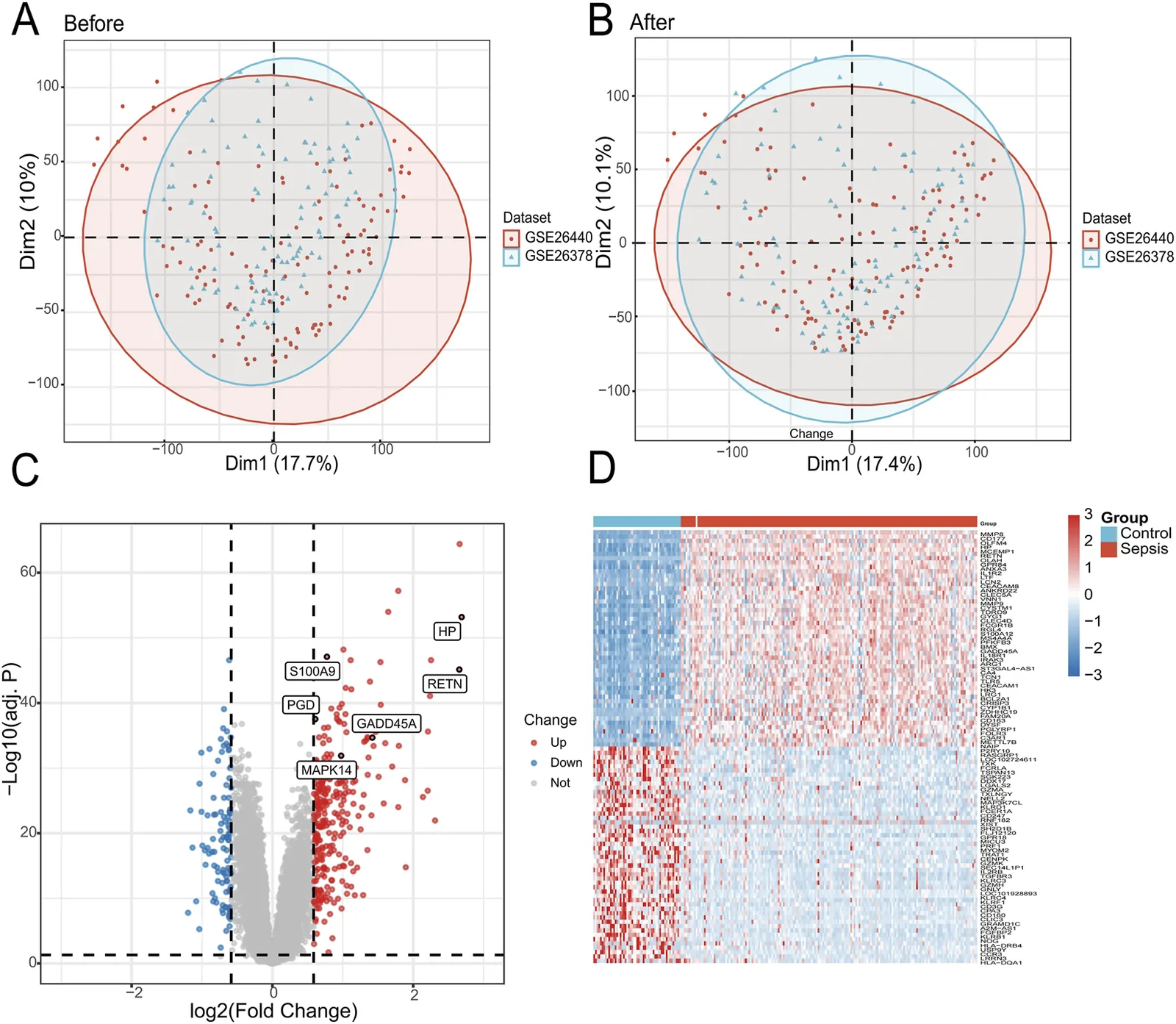
Screening and visualization of differentially expressed genes in pediatric sepsis. **(A,B)** Principal component analysis (PCA) plots describe data before and after batch effects. **(C)** Volcano plot displaying differentially expressed genes (DEGs) between sepsis and control groups in GSE26440 and GSE26378 (red: upregulated; blue: downregulated; gray: non-significant genes). **(D)** Heatmap showing the top fifty DEGs.

### 3.2 GO and KEGG enrichment analysis

To further explore the relationship between DEGs in pediatric sepsis and OSRGs, GO and KEGG enrichment analyses were performed on 36 key OSRGs to elucidate their biological functions. GO analysis revealed significant enrichment in BP associated with OS (Figure 3A). KEGG pathway analysis identified nine pathways implicated in the progression of sepsis, including neutrophil extracellular trap (NET) formation and the regulation of inflammatory mediators affecting TRP channels. Additionally, the Toll-like receptor (TLR) and NOD-like receptor signaling pathways, both of which play essential roles in innate immune responses, were highlighted (Figure 3B). Among these, NET formation was identified as particularly critical in the pathogenesis of sepsis (Figure 3C).

**FIGURE 3:**
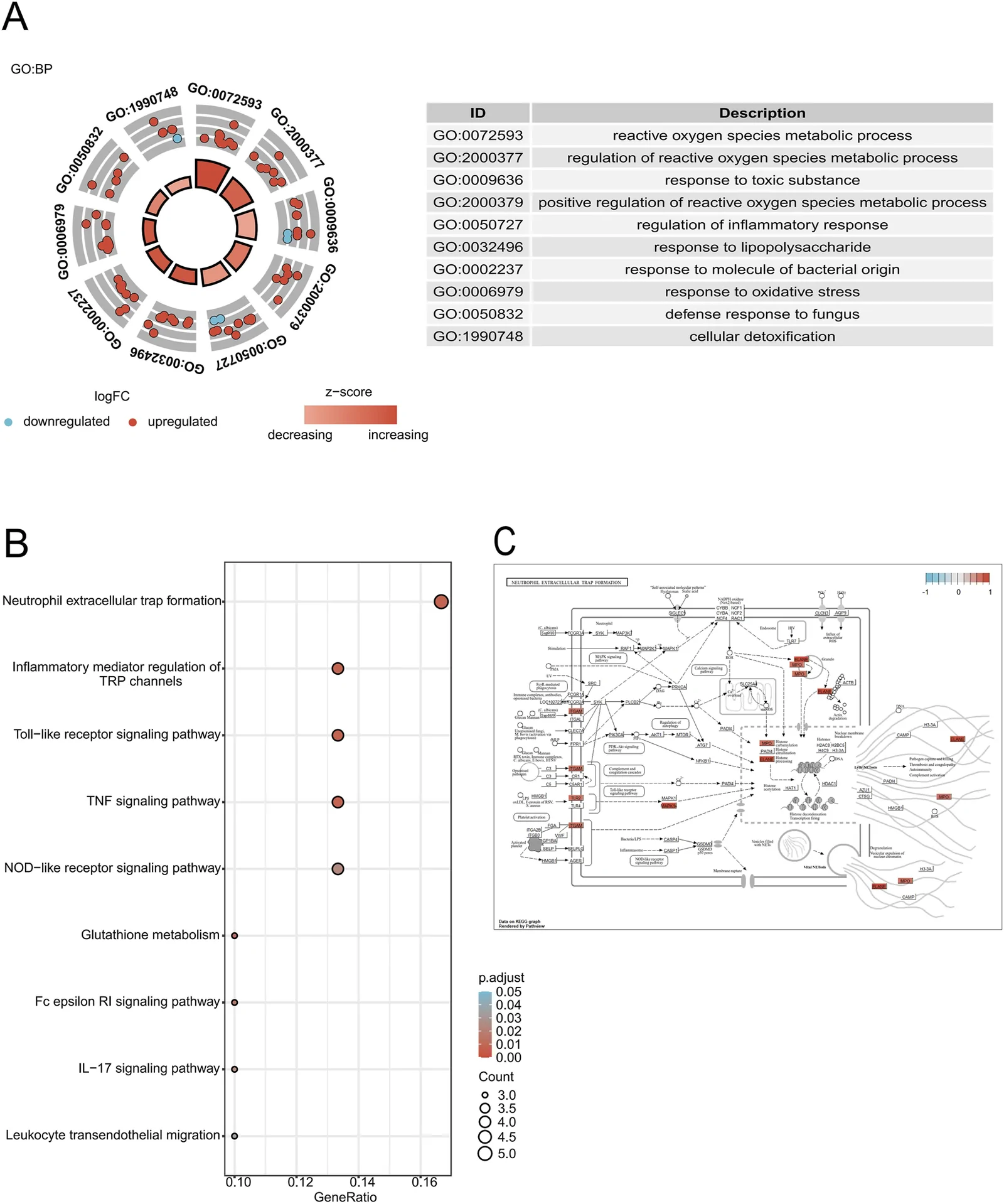
Gene Ontology (GO) and Kyoto Encyclopedia of Genes and Genomes (KEGG) enrichment analysis of oxidative stress (OS) related DEGs. **(A)** GO enrichment analysis of OS-related DEGs involved biological process. **(B)** KEGG enrichment analysis of OS-related DEGs involved signaling pathway. **(C)** NET formation signaling pathway based on the KEGG database.

### 3.3 GSEA of phenotype scores

Our investigation evaluated the role of OSRGs in disease progression by constructing OS phenotypic scores. The OS phenotypic score was significantly higher in the sepsis cohort compared to the control group (P < 0.001; Figure 4A). GSEA of the sepsis cohort revealed upregulation of BP such as “ROS metabolic process” and “response to OS” in the high OS group. Furthermore, KEGG pathway analysis indicated enhanced activation of sepsis-associated pathways in the high OS group. A chord diagram integrating log fold change values further illustrated the key signaling pathways associated with OSRGs (Figures 4B–D). Collectively, these findings provide novel insights into the molecular mechanisms of OS in sepsis and highlight its potential role in disease progression.

**FIGURE 4:**
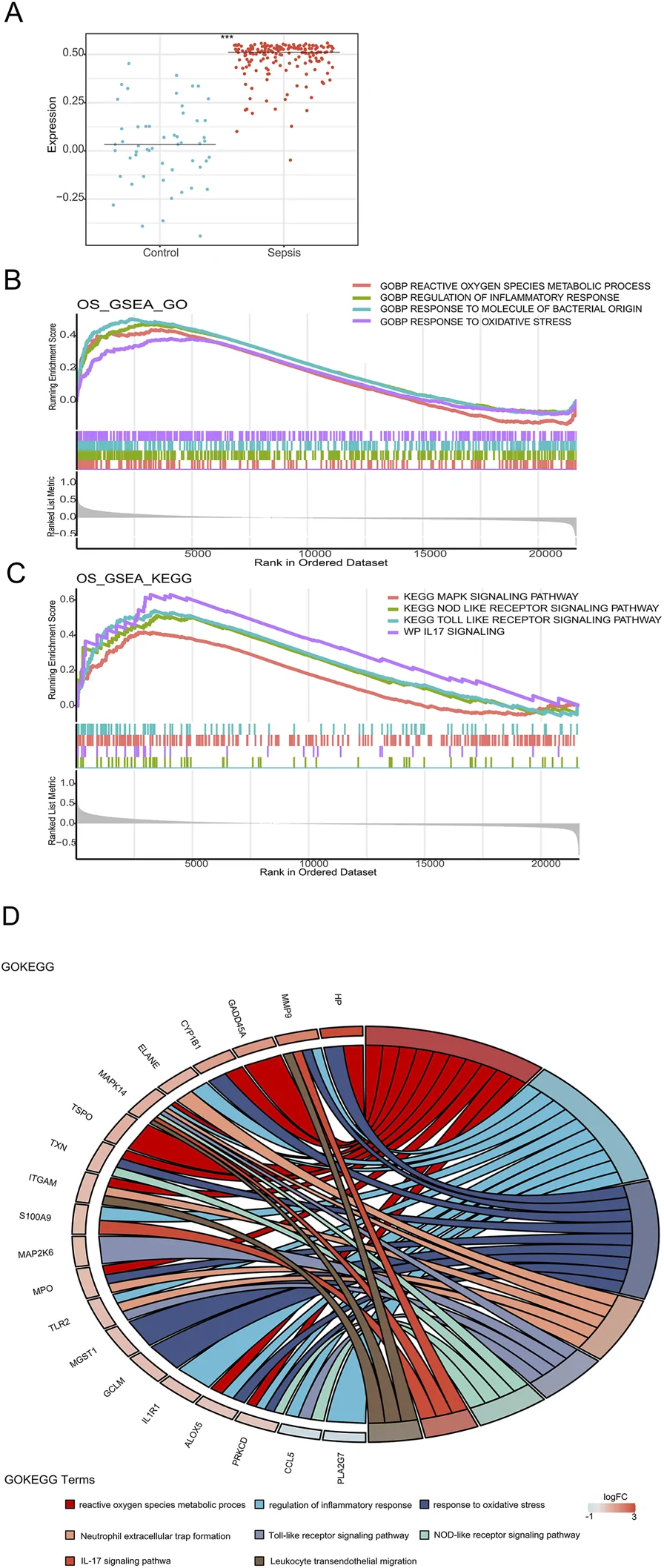
Functional enrichment analysis in phenotype scores of OS. **(A)** Scatter plot of OS phenotype scores in sepsis and normal samples. **(B)** Gene set enrichment analysis (GO) in the high OS group. **(C)** Gene set enrichment analysis (KEGG) in the high OS group. **(D)** A chord diagram of joint log fold change.

### 3.4 Screening and recognition of characteristic differentially expressed OSRGs

To further explore the association between OSRGs and sepsis, WGCNA was applied to identify critical gene modules connected to the disease. Supplementary Figure S7A shows a hierarchical clustering analysis of all samples in the training cohort, confirming that no significant outliers were present. Figure 5A illustrates the selection process for the soft-thresholding power. At a soft threshold of β = 8, the scale-free topology fit index (R^2^) reaches 0.85, indicating that the constructed network closely approximates a scale-free distribution. Using the dynamic tree cut method, genes were clustered, and highly similar modules were merged (Supplementary Figure S7B), resulting in 14 distinct co-expression modules (Figure 5B). Correlation analysis revealed that the “turquoise” module had a strong positive association with sepsis (r = 0.65, p = 4.57 × 10^−29^) and a corresponding negative correlation with controls (r = −0.65, p = 4.57 × 10^−29^), suggesting its pivotal role in disease progression (Figure 5C). Furthermore, a scatterplot analysis of module membership (correlation of each gene with the module eigengene) versus gene significance (correlation of each gene with the disease trait) demonstrated a robust positive correlation within the turquoise module (r = 0.58, p = 1.3 × 10^−90^; Supplementary Figure S7C), indicating that member genes collectively contribute to disease association. Finally, Venn diagram analysis integrating DEGs, OSRGs, and key module genes identified the intersection set of OS–related DEGs in sepsis (Figure 5D).

**FIGURE 5:**
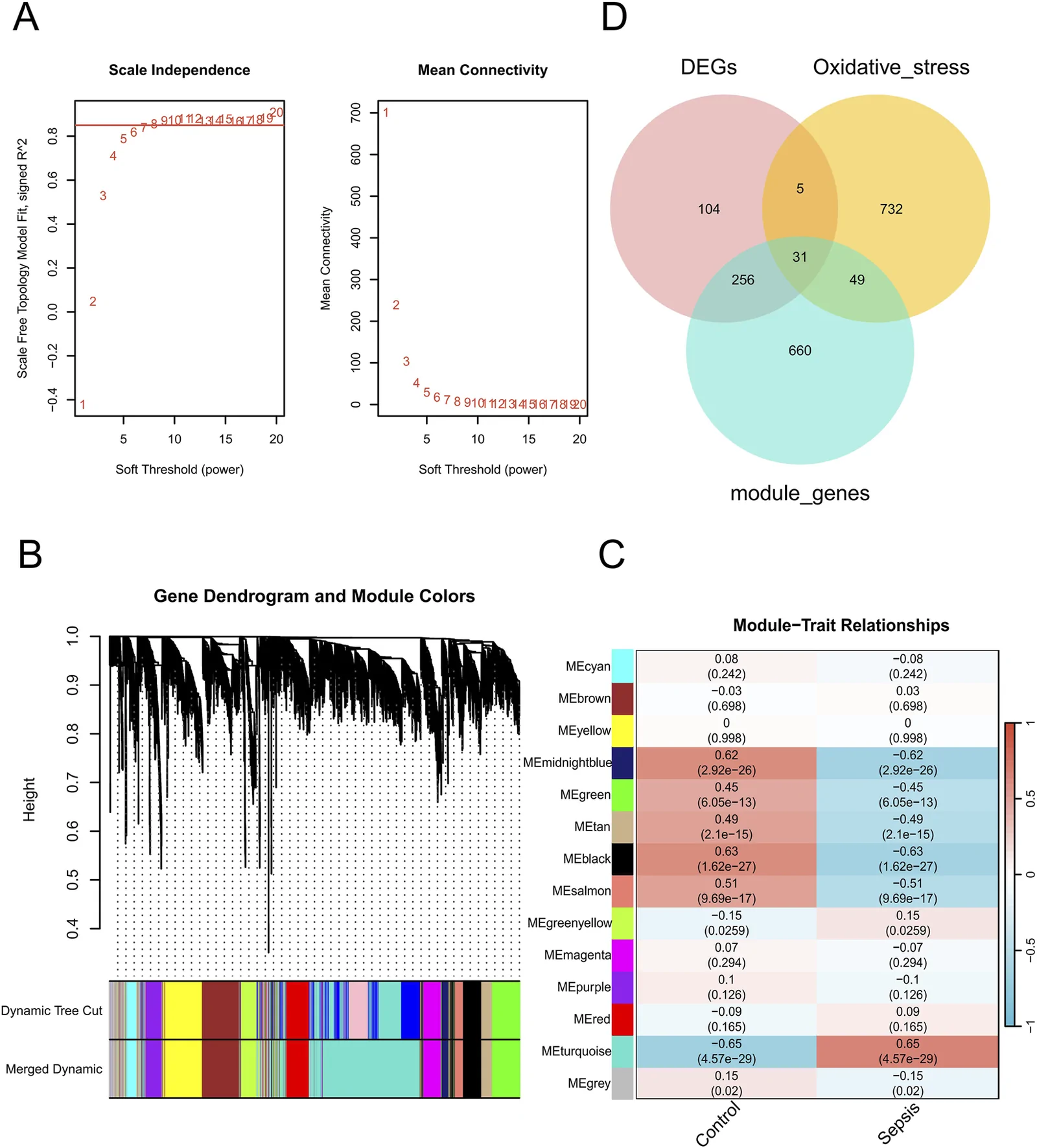
Weighted gene co-expression network analysis (WGCNA) of key module genes. **(A)** Determination the optimal soft threshold (β = 8) for scale-free network construction. **(B)** Clustering dendrogram of genes and merging of similar modules following dynamic tree cutting. **(C)** Heatmap of the correlation between modules and sepsis traits. **(D)** Venn diagram of 31 overlapping genes showing the interactions between DEGs, oxidative stress-related genes (OSRGs), and key module genes.

### 3.5 Identification of candidate genes by multiple ML

ML algorithms have become increasingly important in data science for classification and prediction tasks. In this study, 6 ML algorithms were trained using the R package “caret.” Figure 6 presents the feature screening results associated with various machine learning algorithms. The LASSO algorithm identified 10 OSRGs associated with sepsis: *ALDH1A1, DDAH2, GADD45A, GCLM, HP, MAPK14, MGST1, PGD, RETN* and *S100A9* (Figure 6A). The bagging decision tree algorithm identified 16 OSRGs: *RETN, HP, ARG1, S100A9, GADD45A, DDAH2, PGD, TSPO, LTF, KCNE1, CYP1B1, MAPK14, MMP9, MGST1, IL1RN* and *GCLM* (Figure 6B). The RF algorithm highlighted 19 OSRGs: *RETN, HP, ARG1, S100A9, DDAH2, GADD45A, KCNE1, PGD, LTF, MAPK14, CYP1B1, MGST1, IL1RN, MAP2K6, GCLM, TSPO, F5, ADM* and *ALDH1A1* (Figure 6C). The Bayesian algorithm identified 10 OSRGs: *HP, RETN, DDAH2, KCNE1, TSPO, PGD, GADD45A, S100A9, ARG1* and *MAPK14* (Figure 6D). Using Boruta feature selection (Figure 6E) and the LVQ algorithm (Figure 6F), we obtained a combined set of 31 OSRGs. Since each algorithm uses different importance metrics, some panels illustrate the screening frequency or variable importance score at the gene level, while others depict the cross-validation performance across different feature subsets. These outputs are then used to identify candidate genes from each algorithm. The candidate genes identified by each algorithm are further consolidated, and the final robust candidate genes are established through a consensus approach, such as taking the intersection of multiple algorithms.

**FIGURE 6:**
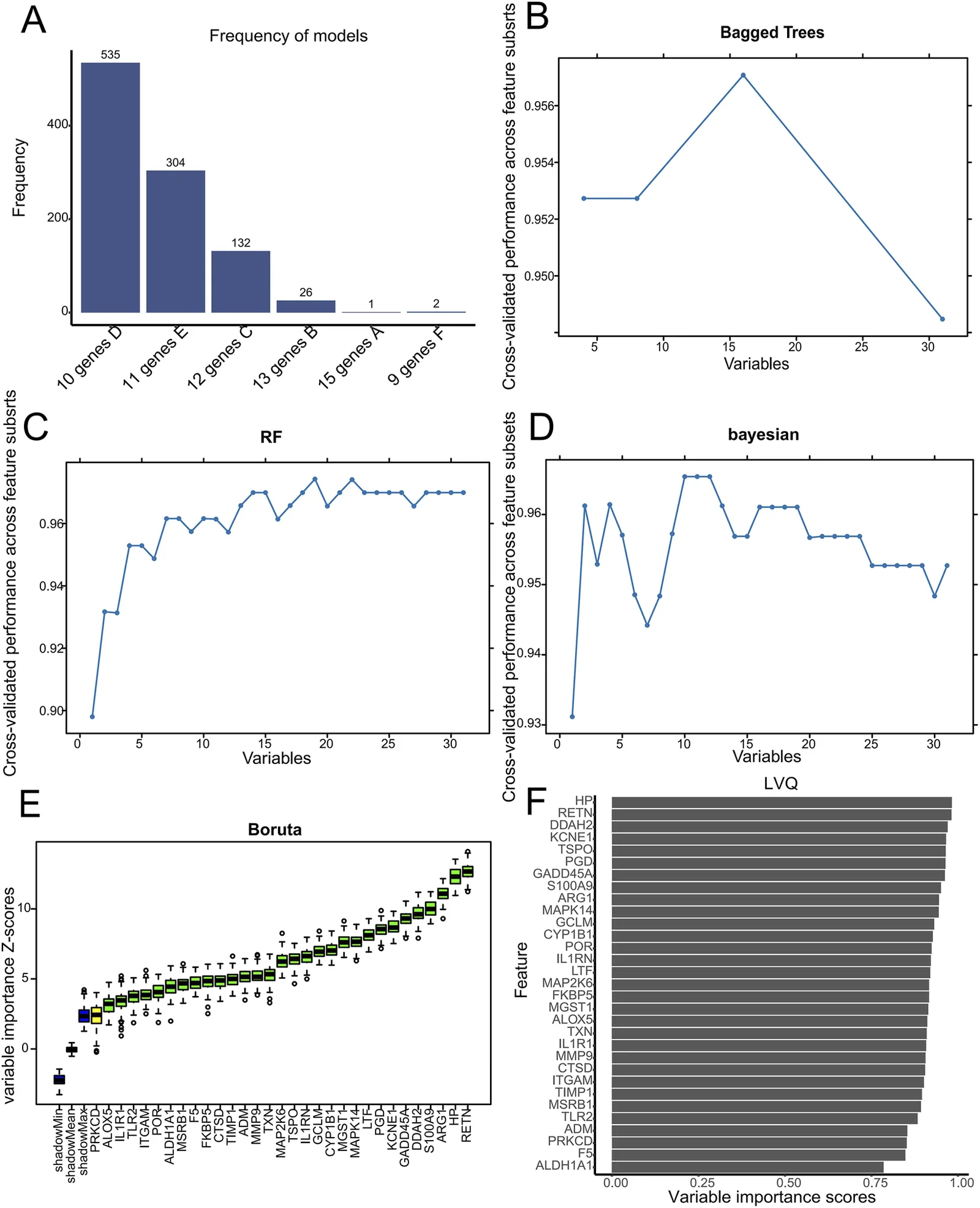
Feature selection outputs from multiple machine learning algorithms. **(A)** LASSO-based feature selection frequency or non-zero coefficient genes. **(B)** Cross-validated performance of bagged trees across different feature subsets. **(C)** Cross-validated performance of random forest across different feature subsets. **(D)** Cross-validated performance of Bayesian model across different feature subsets. **(E)** Boruta variable importance Z-scores. **(F)** Variable importance scores generated by learning vector quantization (LVQ).

To ensure robustness, we integrated the outputs of all six algorithms and identified consensus genes present across all methods. This yielded a final set of seven candidate diagnostic genes for pediatric sepsis: *DDAH2, GADD45A, HP, MAPK14, PGD, RETN,* and *S100A9* (Figure 7A). All the aforementioned candidate genes have satisfied the multi-algorithmic consistency screening criteria and have been identified as candidate gene features using six machine learning methods. This suggests that the screening results are not dependent on any single algorithm. For example, DDAH2 was included among the seven candidate diagnostic genes because it was consistently identified in the candidate results of all six machine learning algorithms, rather than because it ranked higher in any one algorithm.

**FIGURE 7:**
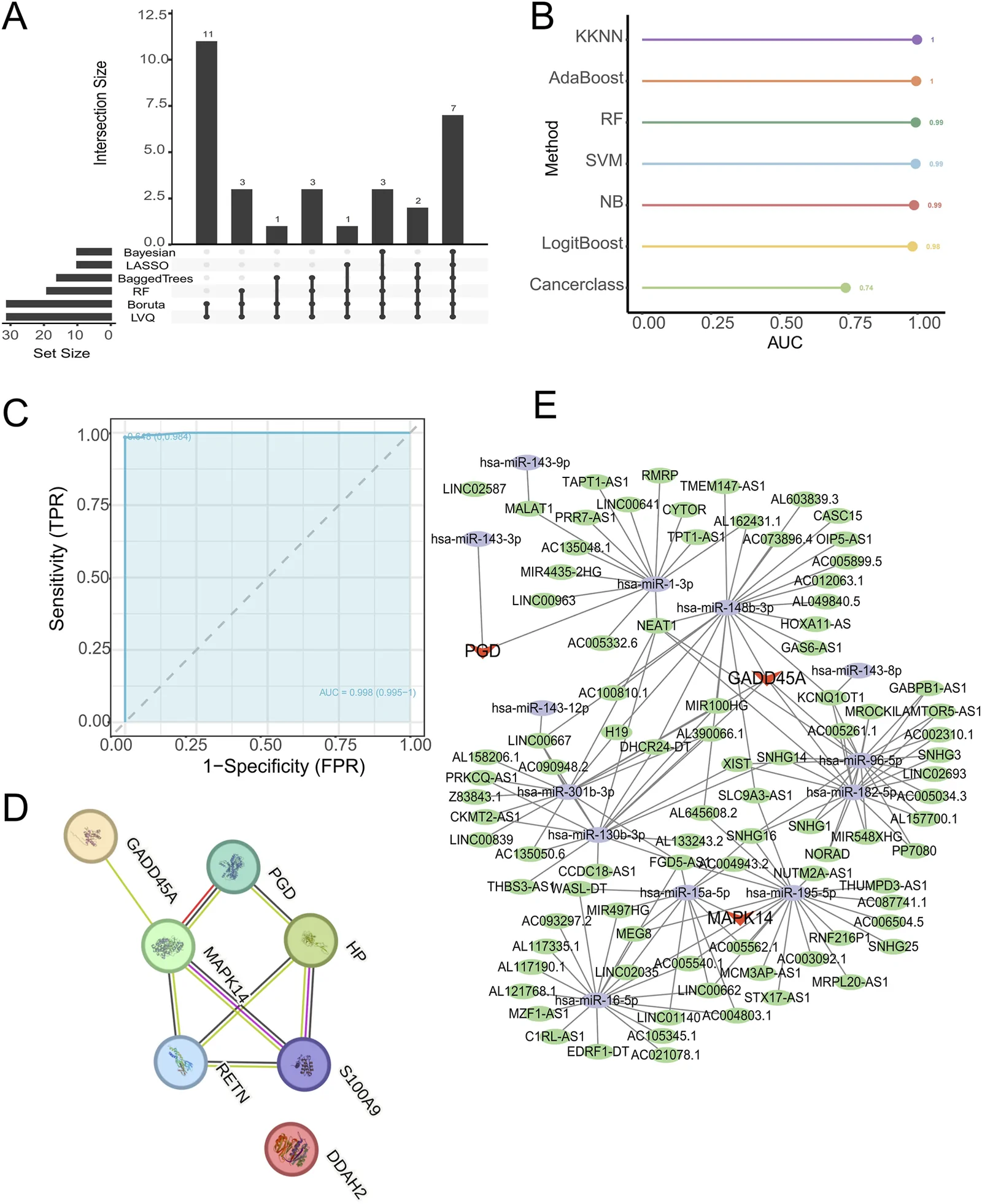
Construction of early diagnosis models by machine learning (ML) algorithms and Protein-Protein Interaction (PPI) network analysis. **(A)** The upset plot visualizes the intersection of candidate genes identified by six machine learning algorithms (final consensus candidate genes defined by the 6/6 intersection criterion). **(B)** Prediction model for pediatric sepsis incidence constructed using 7 ML algorithms and validated on the integrated cohort (GSE13904, GSE8121, and GSE9692). **(C)** Visualization of the ROC curve generated by the K-NN algorithm for the prediction of pediatric sepsis occurrences. **(D)** PPI network analysis for establishing six hub oxidative stress-related genes interaction. **(E)** mRNA-miRNA-lncRNA regulatory network based on six hub oxidative stress-related genes.

### 3.6 Early prediction model construction for pediatric sepsis

To investigate the relationship between OSRGs and the development of pediatric sepsis, we employed 7 ML algorithms to construct predictive models using the pooled training cohorts (GSE26440 and GSE26378). The optimal predictive model was determined by comparing the AUC values across the algorithms. The algorithms included RF, SVM, LogitBoost, k-NN, NB, AdaBoost Classification Tree (AdaBoost), and CancerClass, an algorithm designed for classification analysis. Following model selection, the pooled validation cohorts were used to further evaluate diagnostic performance, and AUC values were compared among models (Figure 7B). The results demonstrated that the predictive model constructed using the k-NN algorithm exhibited superior diagnostic accuracy compared to the other approaches (Figure 7C). In the independent validation cohort, seven models were compared based on their AUC values. Among them, k-NN was selected as the final model due to its best AUC performance in the validation cohort. Among the seven candidate classifiers, the CancerClass model achieved a lower AUROC on the validation set than the other algorithms. This finding suggests that this classification strategy has limited adaptability to the specific combinations of candidate genes and the expression data related to sepsis in children used in this study. In contrast, the k-NN model achieved the highest AUROC in the validation set. Therefore, it was selected as the final prediction model. This performance evaluation was conducted using a validation cohort that did not engage in feature screening or model parameter tuning; therefore, the validation set labels were not utilized in the prior candidate gene screening process.

### 3.7 Identification the interactions of hub OSRGs

To investigate gene–gene interactions, we constructed a PPI network using the STRING database, based on seven candidate genes identified across 6 ML algorithms. Isolated nodes were excluded to focus on biologically meaningful connections, which resulted in the identification of six hub genes: S100A9, HP, PGD, MAPK14, GADD45A and RETN (Figure 7D).

To further investigate post-transcriptional regulation, we developed a ceRNA network to examine the role of lncRNAs in modulating miRNA activity in pediatric sepsis. The network integrated upregulated lncRNAs with co-expressed OSRGs and predicted miRNA interactions (Figure 7E). The final ceRNA network consisted of 102 nodes and 160 edges, including three hub OSRGs, 89 lncRNAs, and 10 miRNAs. Detailed analysis revealed that 85 lncRNAs competitively bound to five miRNAs regulating GADD45A expression. Within the MAPK14-centered ceRNA subnetwork, we identified 48 lncRNAs interacting with three miRNAs to regulate MAPK14 and its target genes. Similarly, 17 lncRNAs competed with two miRNAs to modulate PGD expression, indicating a multilayered ceRNA-mediated regulatory mechanism underlying sepsis progression. The constructed mRNA-miRNA-lncRNA network, based on hub genes, indicates that GADD45A, MAPK14, and PGD may establish candidate regulatory relationships with several predicted miRNAs and lncRNAs. This finding implies that hub genes associated with oxidative stress could be regulated post-transcriptionally at the level of non-coding RNAs; however, the identified miRNA and lncRNA nodes primarily originate from database predictions. Additional experiments are necessary to confirm the expression changes and to elucidate their direct relationship with sepsis in children.

### 3.8 Alterations in peripheral blood immune cell composition

The deconvolution analysis of peripheral blood immune cells using CIBERSORT indicated distinct relative proportions of immune cells between the sepsis group and the control group, implying that pediatric sepsis is associated with alterations in peripheral blood immune cell composition rather than immune cell infiltration at the tissue level. The stacked bar chart depicts the relative distribution of immune cell subtypes (Figure 8B), revealing marked alterations in neutrophils, CD8 T cells, monocytes, and M2 macrophages within the sepsis cohort. Specifically, the proportions of M0 macrophages, M2 macrophages, monocytes, activated mast cells, and neutrophils were significantly elevated in sepsis patients (P < 0.001), whereas follicular helper T cells, CD8 T cells, CD4 memory T cells, resting dendritic cells, and activated NK cells were markedly reduced (P < 0.001) (Figure 8A). Correlation heatmap analysis demonstrated strong interactions among immune subtypes: M2 macrophages positively linked with neutrophils, whereas activated NK cells had negative correlations with both neutrophils and mast cells (Figure 8C). Furthermore, the correlation chord diagram highlighted associations among hub genes (Figure 8D). Correlation lollipop chart revealed that most hub OSRGs were positively correlated with neutrophils and negatively linked with resting dendritic cells (Figures 8E–J). Collectively, these results propose that hub OSRGs may be involved in triggering and advancing sepsis by modulating the abundance and activity of distinct immune cell subsets in the immune microenvironment.

**FIGURE 8:**
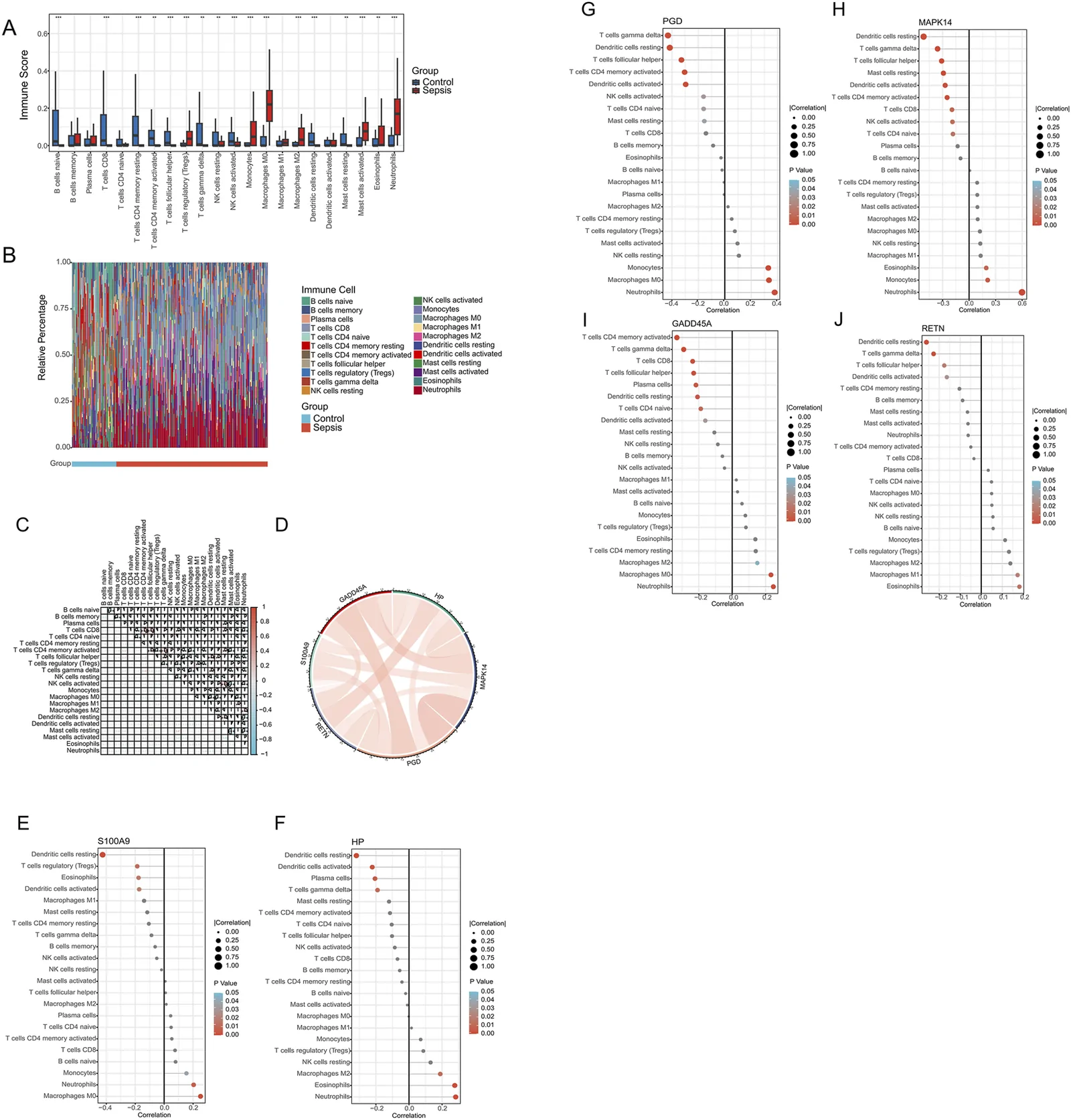
Immune cell composition analysis utilizing the CIBERSORT algorithm and correlation with hub OSRGs in sepsis. **(A)** Differential composition of 22 immune cell types between control (blue) and sepsis (red) groups. **(B)** Stacked bar chart of 22 immune cell types in control and sepsis groups. **(C)** Correlation heatmaps of different immune cell infiltration. **(D)** Correlation chord diagram of hub OSRGs. **(E–J)** Correlation lollipop plots showing associations between each hub OSRG and immune cell subsets. **(E–J)** Correlation lollipop chart analysis between S100A9 **(E)**, HP **(F)**, PGD **(G)**, MAPK14 **(H)**, GADD45 **(I)** and RETN **(J)** with immune cell types.

### 3.9 Diagnostic performance evaluation based on OS-related biomarkers

To assess the diagnostic performance of hub OSRGs in pediatric sepsis, we established a diagnostic model including six hub genes recognized via ML and PPI analysis. Expression levels of S100A9, HP, PGD, MAPK14, GADD45A and RETN were significantly elevated in the sepsis group compared to controls. ROC curve analysis revealed high diagnostic accuracy, with AUC values of 0.951, 0.982, 0.964, 0.944, 0.962, and 0.982, respectively (Figures 9A–F). A nomogram constructed using these six genes achieved an AUC of 0.994 (Figures 9G,H), underscoring the strong predictive power of the model. Validation set analysis confirmed consistent results: the six genes remained significantly upregulated in sepsis patients, and ROC curves yielded AUC values of 0.914, 0.911, 0.948, 0.908, 0.963, and 0.977, respectively (Supplementary Figure S8). Collectively, these results indicate that GADD45A, S100A9, PGD, MAPK14, HP, and RETN may represent potential oxidative stress–related biomarkers for pediatric sepsis.

**FIGURE 9:**
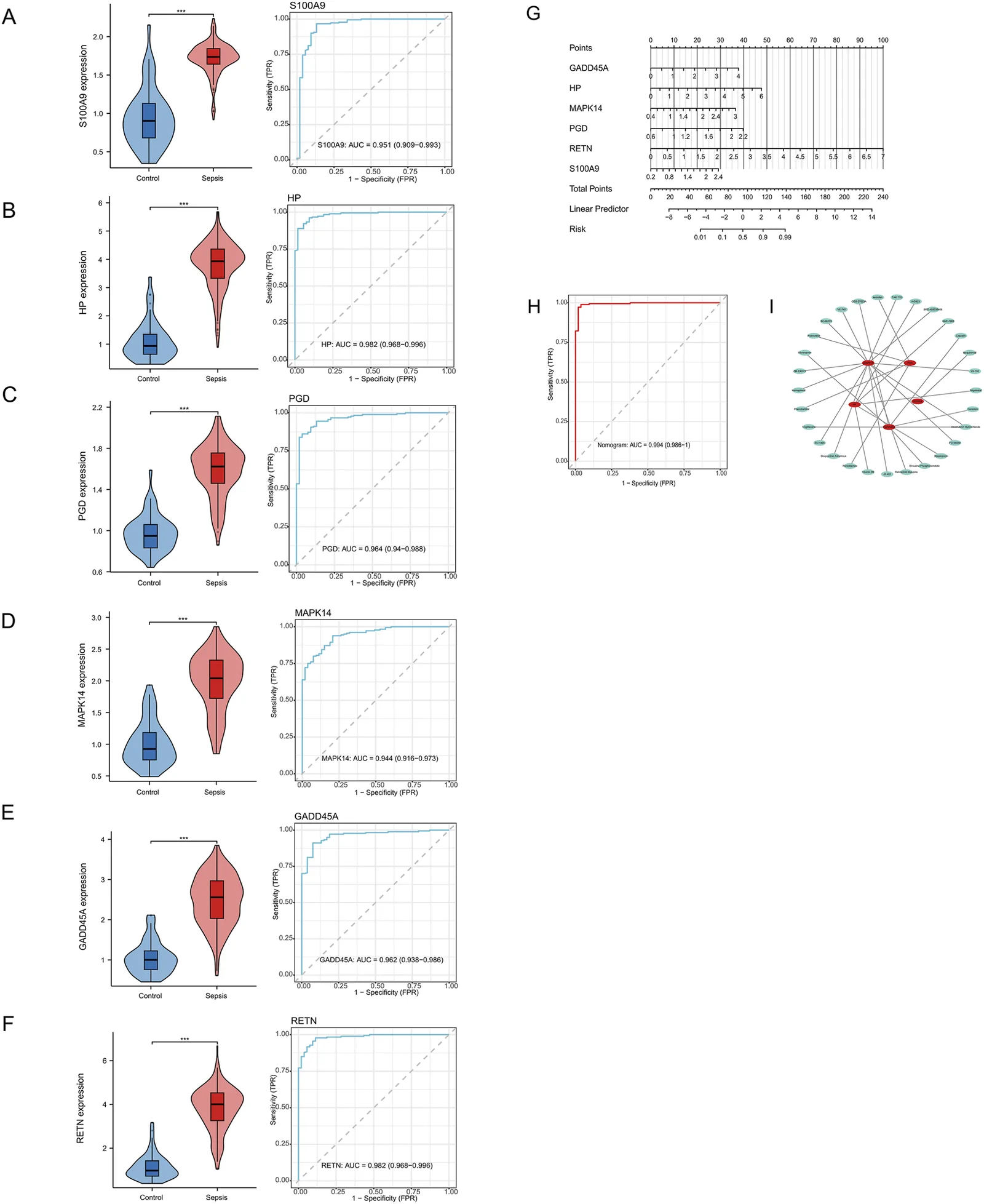
Diagnostic potential of OS-related biomarkers in pediatric sepsis. **(A–F)** Differential expression in control and sepsis group and ROC curve of S100A9 **(A)**, HP **(B)**, PGD **(C)**, MAPK14 **(D)**, GADD45A **(E)** and RETN **(F)**. **(G)** Nomogram model predicated on the six hub genes. **(H)** ROC curve of the Nomogram model. **(I)** Interaction network of six hub genes and small molecule compounds.

### 3.10 Molecular docking and drug prediction

To enhance the clarity of the candidate compound screening process, this study organizes the primary steps of drug prediction and molecular docking into flowcharts and summarizes the key screening results (Supplementary Figure S9; Supplementary Table S3). The screening process begins with hub oxidative stress-related genes and integrates data from DGIdb and cMap to identify candidate small molecules. Subsequently, the potential binding affinity of these compounds to target proteins is evaluated using CB-Dock2. The final candidate results are compiled based on database sources, corresponding target genes, and docking scores, thereby illustrating the rationale for each compound’s inclusion in molecular docking and subsequent retention. The gene-drug interaction network revealed a range of drug-like compounds with potential sensitivity toward key target proteins (Figure 9I). Based on disease-associated hub genes, the cMap database was further used to screen candidate compounds predicted to interact with hub gene-encoded proteins. Compounds showing negative correlations with disease-related protein expression were prioritized. Notably, MAPK14 was associated with doxycycline anhydrous and ralimetinib mesylate, while S100A9 was linked to tasquinimod. To validate these findings, blind molecular docking of hub OSRGs with the candidate compounds was performed using the CB-Dock2 platform. The MAPK14 protein demonstrated strong binding affinities for doxycycline and ralimetinib mesylate, with AutoDock Vina scores of −8.4 and −8.1 kcal/mol, respectively (Figures 10A,B). Doxycycline interacted with MAPK14 through multiple ionic bonds, hydrophobic forces, hydrogen bonds, and weak hydrogen bonds involving residues such as PRO350, VAL349, PHE348, SER347, ILE346, VAL345, TYR342, SER95, ARG94, ALA93, PRO92, THR91, PHE90, VAL89, ASP88, LEU87, LYS45, TYR24, ARG23, PRO21, PHE8, PRO6, ARG5. In contrast, ralimetinib mesylate primarily engaged MAPK14 through hydrogen bonding, hydrophobic forces, ionic interactions, and cation–π interactions, involving residues including GLU328, ASP205, TYR200, ILE193, ARG189, THR185, ALA184, GLY170, PHE169, ASP168, LEU167, ILE166, ASN155, SER154, LYS152, ASP150, ARG149, HIS148, ILE147, ILE146, ILE141, ASP112, LEU108, THR106, ILE84, VAL83, MET78, LEU75, LEU74, GLU71, ARG70, THR68, ARG67, LYS66, LEU55, LYS53, VAL38, GLY36, TYR35, ALA34, GLY33, SER32, GLY31, VAL30. Similarly, the S100A9 protein exhibited a significant binding affinity for tasquinimod, with a docking score of −7.0 kcal/mol (Figure 10C). Tasquinimod engaged S100A9 via hydrogen bonding, hydrophobic forces, and π–π interactions, involving residues MET94, HIS91, SER90, TRP88, THR87, LEU86, ALA84, MET83, LEU82, ILE80, PHE79, PHE48, ASN47, GLN46, LEU45, ASP44, LEU40, GLN21, PHE19, THR18, ASN17, ILE15, THR14, ILE12, ASN11, LEU8, GLN7. Collectively, these results highlight MAPK14 and S100A9 as promising therapeutic targets, with doxycycline, ralimetinib mesylate, and tasquinimod emerging as candidate small-molecule modulators for pediatric sepsis.

**FIGURE 10:**
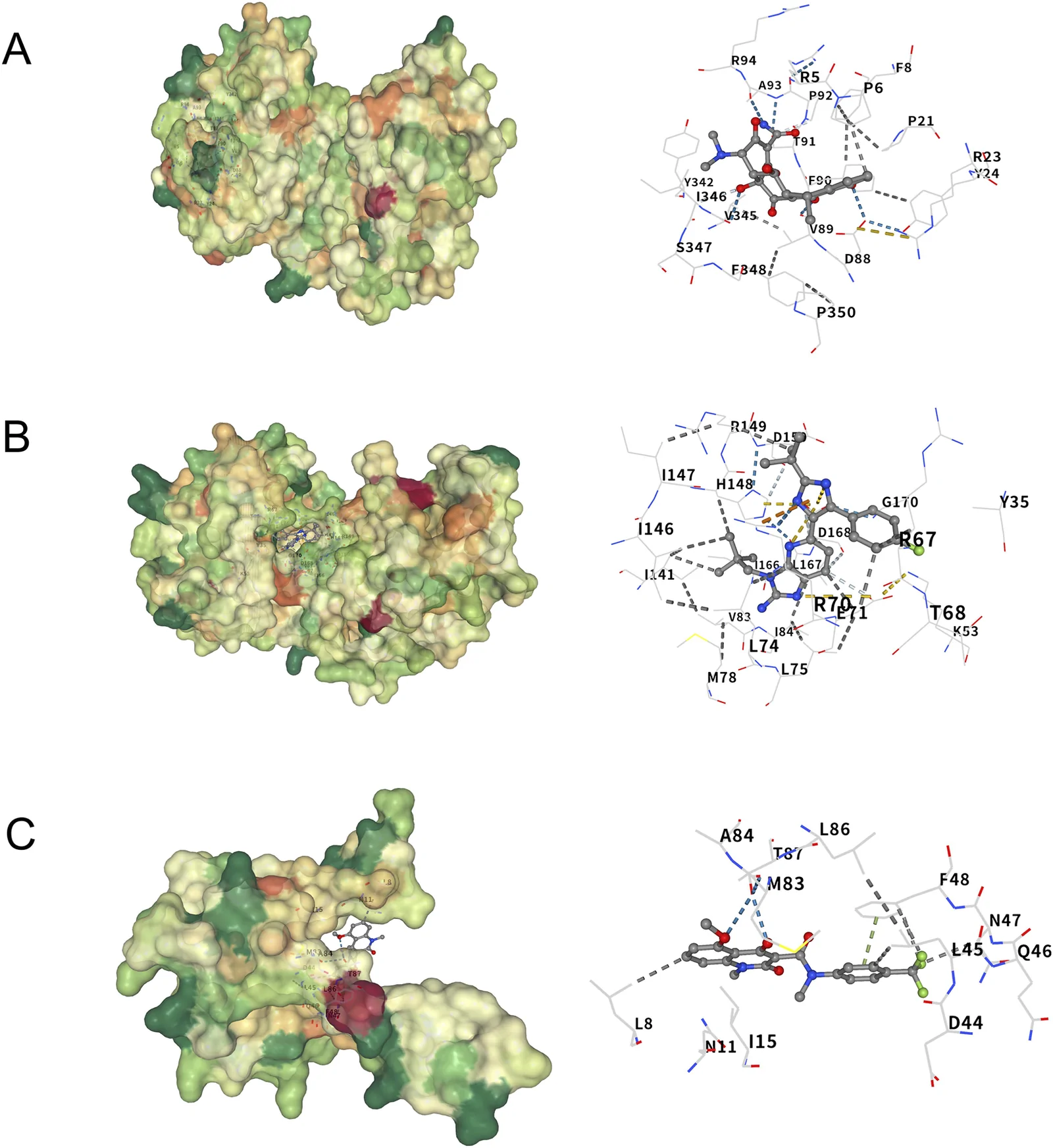
Visualization the result of Molecule Docking. **(A)** MAPK14 with Doxycycline. **(B)** MAPK14 with Ralimetinib mesylate. **(C)** S100A9 with Tasquinimod.

## 4 Discussion

This study demonstrates the clinical significance of identifying differentially expressed OSRGs in sepsis. The multi-gene diagnostic model developed in this study, comprising GADD45A, MAPK14, HP, PGD, RETN, and S100A9 as core components, demonstrated favorable performance in distinguishing sepsis from healthy controls. Compared with conventional clinical biomarkers such as procalcitonin (PCT), C-reactive protein (CRP), and lactate, this gene signature may more sensitively reflect early molecular alterations associated with oxidative stress and immune dysregulation, thereby providing complementary diagnostic information during the early stages of disease progression (Florin et al., 2020). Moreover, the integration of multiple molecular indicators is expected to complement existing biochemical markers, thereby improving the specificity and overall predictive accuracy of the diagnostic model. This advantage is particularly relevant in cases where conventional indicators are inconclusive or clinical manifestations are atypical, suggesting potential translational and clinical utility. Future prospective studies are warranted to further evaluate the additive or synergistic effects of combining these molecular markers with established clinical parameters and to assess their feasibility in routine diagnostic workflows (Zhang et al., 2023). The primary aim of this study is to identify oxidative stress-related diagnostic features within the pediatric sepsis transcriptome cohort and to assess their stability across independent pediatric cohorts. Published biomarker signatures for adult sepsis may be influenced by factors such as the source of the adult cohort, disease severity, timing of sample collection, and model construction strategy. Therefore, their applicability to the pediatric population requires further validation through a standardized analytical approach. A recent study on pediatric sepsis biomarkers utilizing RNA-seq data established a significant reference for the field. Ye et al. integrated six RNA-seq datasets pertaining to childhood sepsis and employed Weighted Gene Co-expression Network Analysis (WGCNA) alongside 14 machine learning models to identify 237 high-confidence biomarkers. Notably, RORA and GPR183 exhibited substantial feature contributions across multiple models. The area under the curve (AUC) for the majority of the identified biomarkers surpassed 0.7 (Ye et al., 2026). In contrast to this study, the candidate gene set in this study was restricted to genes associated with oxidative stress, identifying GADD45A, HP, MAPK14, PGD, RETN, and S100A9 in the pediatric sepsis microarray cohort. The hub genes identified among oxidative stress-related genes in this study are implicated in reactive oxygen species (ROS) metabolism, mediate inflammatory responses and immune imbalances, and activate the MAPK/TLR/NOD-like receptor-related inflammatory pathways, thereby contributing to the onset and progression of sepsis in children. Furthermore, regarding diagnostic performance, the area under the curve (AUC) values for individual genes ranged from 0.908 to 0.977. This result further supports the potential of the six hub oxidative stress-related genes as biomarkers for predicting pediatric sepsis. Although both studies highlighted the potential application value of transcriptomic features in diagnosing sepsis in children, the differences in sequencing platforms, candidate gene screening, data integration methods, and machine learning techniques made the AUC values reported in each study impossible to directly compare. As a result, these values could only serve as indirect references. Previous studies have described the role of OS in children with sepsis and examined strategies to reduce inflammatory injury by targeting OS-related pathways (von Dessauer et al., 2011). Our findings further confirm that hub genes are implicated in critical BP regulating immune responses and inflammation, both of which undergo significant alterations as sepsis progresses (Macdonald et al., 2003; Arina and Singer, 2021). Upregulation of hub OSRGs is associated with elevated OS, thereby intensifying local tissue injury and amplifying the systemic inflammatory response (Lu et al., 2022). Collectively, these findings deepen the understanding of OS mechanisms and their clinical implications in sepsis and lay the foundation for future therapeutic interventions.

GO enrichment analysis revealed that OSRGs participate in essential BP, including ROS metabolism and inflammation, both of which enhance immune activity and represent potential therapeutic targets (Prauchner, 2017). GSEA further highlighted the central role of OS in sepsis pathophysiology. Additionally, KEGG enrichment analysis emphasized not only the interplay between ROS and immune responses in sepsis but also potential pharmacological pathways for mitigating harmful oxidative reactions. Of particular note, sepsis markedly enriched the NET signaling pathway, indicating that NET formation contributes to disease progression. Neutrophils, as pivotal components of the innate immune system, combat infections by generating NETs, DNA and antimicrobial protein structures designed to capture and eliminate extracellular pathogens. NET formation relies on ROS generated by NADPH oxidase (Castanheira and Kubes, 2019). Since the discovery of their network-like antimicrobial structure in 2004, research has primarily focused on the pathogen-trapping and protective roles of NETs (Brinkmann et al., 2004). However, in sepsis, an excessive immune response drives uncontrolled NET production, aggravating sepsis-associated lung injury (Bukong et al., 2018). Pathway analyses revealed that OSRGs are highly expressed and upregulated during NET formation. The IL-17 signaling pathway was found to recruit neutrophils and stimulate NET formation (Zhang et al., 2020). Glutathione, a critical intracellular antioxidant, plays a key role in cellular defense, and its depletion intensifies OS by promoting ROS accumulation and inflammation (Ghezzi, 2011). De Backer et al. employed orthogonal polarization spectral (OPS) imaging to study sublingual microcirculation in septic patients, reporting for the first time in humans that sepsis alters microcirculatory blood flow and reduces vascular perfusion (De Backer et al., 2002). These microcirculatory disturbances, driven by endothelial dysfunction, persist throughout septic shock. During sepsis, an imbalance between antioxidant defenses and ROS contributes to mitochondrial dysfunction and further disrupts endothelial integrity. With the involvement of TLRs, endothelial cells acquire a pro-inflammatory, pro-coagulant, and adhesive phenotype (Joffre and Hellman, 2021). A chord diagram was used to illustrate associations between key pathways and OSRGs. Phenotype scoring via GSEA indicated that sepsis-induced organ damage is mediated through activation of the MAPK, TLR, and NOD-like receptor signaling pathways. TLRs, as the body’s frontline defense against invading pathogens, recognize pathogen-associated molecular patterns (PAMPs) and activate innate immune responses (Akira et al., 2006). All Toll-like receptor signaling pathways activate the transcription factor NF-kappaB, which controls the expression of inflammatory cytokines, including TNF, IL-6, IL-8, and type I interferons (Kawai and Akira, 2007). Similarly, NLRP3, a multiprotein inflammasome assembled by NOD-like receptors, is activated when microbial invasion triggers host immune recognition (Broz and Dixit, 2016). ROS generated during sepsis not only damage tissues and organs but also promote NLRP3 activation, leading to the release of IL-18 and IL-1β (Bergsbaken et al., 2009; Zhao et al., 2020). Consequently, targeting NLRP3 inflammasome activation has been proposed as a promising therapeutic approach for inflammatory diseases. For instance, Juan Tang et al. demonstrated that regulating NLRP3 activation can effectively mitigate endotoxemia progression (Tang et al., 2020). Additionally, studies reveal that excessive ROS production activates the MAPK inflammatory signaling cascade, which in turn stimulates NF-κB and upregulates pro-inflammatory mediators (e.g., IL-1β, iNOS, and TNF-α) thereby amplifying ROS-induced inflammation (Liu et al., 2020). Collectively, these findings indicate that the inflammatory response in sepsis is closely linked to OS, and hub genes identified through network analysis may serve as reliable predictors of sepsis onset.

In light of these findings, we propose that hub genes play a pivotal role in sepsis development by upregulating the MAPK, TLR, and NOD-like receptor signaling pathways. Molecular docking results further confirmed that small molecules can inhibit the expression of proteins encoded by these target genes. This evidence underscores the therapeutic potential of modulating hub genes, consistent with prior studies demonstrating that inhibition of inflammatory signaling pathways can mitigate diverse disease processes. For instance, in rheumatoid arthritis (RA), suppression of the MAPK/ERK pathway has been shown to significantly reduce inflammatory responses (Yan et al., 2024). Similarly, in diabetic nephropathy (DN), baicalin effectively inhibits the activation of major pro-inflammatory pathways, including members of the MAPK family such as Erk1/2, JNK, and p38 (Ma et al., 2021). Moreover, research by Sung-Chun Tang et al. revealed that Toll-like receptor (TLR4) signaling pathway enhances neuronal susceptibility to amyloid beta-peptide (Aβ) and OS in Alzheimer’s disease, thereby positioning TLR4 as a promising therapeutic target (Tang et al., 2008). Collectively, our results indicate that targeting cellular signaling pathways to regulate inflammatory mediators may represent an effective therapeutic strategy. In future work, we aim to integrate OS-induced inflammatory mechanisms to further elucidate the contributions of MAPK, TLR, and NOD-like receptor pathways in sepsis. From the perspectives of network topology and functional enrichment analysis, several key interaction modules are closely associated with inflammatory signal transduction and oxidative stress responses. For instance, certain upregulated lncRNAs may attenuate their inhibitory effects on target genes such as GADD45A, MAPK14, and PGD by competitively binding shared miRNAs, thereby enhancing the expression of these genes within inflammatory amplification circuits. This regulatory axis may further influence biological processes including reactive oxygen species (ROS) production, NF-κB pathway activation, and apoptosis. In addition, ceRNA subnetworks involving MAPK14 and GADD45A may contribute to the regulation of immune cell activity, highlighting the importance of post-transcriptional regulation in maintaining immune homeostasis. Moreover, several lncRNAs within this regulatory network have been implicated in immune modulation and tissue repair across various inflammatory conditions, suggesting that they may exert comparable functions in sepsis (Zhuang et al., 2023; Hao and Wei, 2022). Collectively, the constructed ceRNA network reveals complex regulatory interactions between oxidative stress–related genes and non-coding RNAs, providing novel molecular insights into the interplay between oxidative stress and immune dysregulation in sepsis. These findings also establish a theoretical basis for subsequent functional validation and the identification of potential therapeutic targets. The ceRNA network offers preliminary insights into the potential post-transcriptional regulation of hub oxidative stress-related genes. However, since this study did not independently validate the expression or perform functional experiments to corroborate the predicted miRNAs and lncRNAs, these non-coding RNA nodes should not be directly regarded as confirmed sepsis-related molecules in children. Their biological significance requires further validation through subsequent research.

WGCNA facilitated the identification of important sepsis-associated gene modules. The strong correlation observed between the “turquoise” module and sepsis highlights a significant relationship among the identified genes, suggesting potential diagnostic value. This disease-focused approach not only generates extensive data to deepen our understanding of complex sepsis pathophysiology but also supports the development of personalized treatment strategies (Martinez-Paz et al., 2023). We identified modules specifically related to pediatric sepsis and overlapped them with OSRGs, further reinforcing the association between OS and disease progression. Thus, the application of network-based methods provides novel insights into the pathogenesis of pediatric sepsis while simultaneously laying the groundwork for future research on its diagnosis and treatment.

ML offers powerful capabilities to predict and classify unseen data by systematically training, optimizing, and evaluating models. It has become an essential tool for uncovering disease mechanisms and developing preventive strategies, making it highly valuable in bioinformatics. Previous studies have shown that ML can predict diseases by analyzing gene expression profiles; however, limited research has focused on identifying OS-related genes in pediatric sepsis. In this study, we applied ML algorithms to analyze 31 OSRGs significantly associated with sepsis and successfully extracted seven promising candidate genes. Different machine learning algorithms exhibit varying performance with respect to sample size, feature dimensions, and class boundaries. The suboptimal predictive performance of CancerClass model in the present dataset suggests the instability of relying on a single algorithm for the transcriptome data associated with pediatric sepsis. Therefore, evaluating multiple algorithms can facilitate the identification of predictive models better aligned with the current data structure. The good predictive performance of K-NN may be attributed to several factors: the final input features are relatively few, the candidate genes exhibit strong discriminative power between groups, the sample size is limited, and the local sample similarity measure may be more robust to overfitting than the complex nonlinear boundaries learned by other models. Therefore, in the case of limited data sample size, complex models such as random forests and support vector machines may not outperform K-NN, and the model performance still needs to be verified in larger-scale prospective cohorts. This integration of ML substantially enhanced the accuracy of predictive modeling for pediatric sepsis and opened new avenues for clinical application. While ML enables multidimensional analysis of biological datasets, challenges remain regarding data variability and the translation of computational findings into clinical practice (Binson et al., 2024). This article presents the methods as a methodological background, without implying their direct applicability to the current data volume and research objectives. In line with this, the present study has not employed deep learning or large-model analysis. Future investigations may explore larger-scale, multi-center, or multi-omics data. Nevertheless, the candidate genes identified here may accelerate the discovery of novel biomarkers and the development of innovative diagnostic tools, thereby complementing existing clinical approaches.

The study of immune cell deconvolution analysis adds an additional layer of complexity to understanding the interplay between OS and sepsis by uncovering significant alterations in cellular composition. Notably, the populations of M0 macrophages and neutrophils were markedly increased and showed positive correlations with the expression of OSRGs, highlighting important immunological implications for both disease progression and therapeutic strategies. Fan Gao et al. reviewed the interactions between NETs and immune cells, emphasizing the critical role of NETs in shaping immune responses and driving disease, while also discussing therapeutic strategies that target NETs to provide new clinical insights (Gao et al., 2025). Furthermore, the TLR family, expressed on neutrophils, macrophages, and dendritic cells, functions as a key group of pattern recognition receptors (PRRs), initiating innate immune responses and bridging them to adaptive immunity (Iwasaki and Medzhitov, 2004). However, in the context of sepsis, excessive activation of immune cells during the acute inflammatory phase often transitions into an immunosuppressive state, predisposing patients to persistent secondary infections and elevating the risk of long-term mortality (Nedeva, 2021). These observations suggest that immunomodulation may be an essential therapeutic approach to mitigate the detrimental consequences of hyperactive immune responses in sepsis (Boomer et al., 2014). Accordingly, targeted interventions designed to restore immune homeostasis could represent a valuable addition to current sepsis management strategies.

## 5 Limitations and future directions

Firstly, this analysis is primarily based on publicly available GEO transcriptomic datasets. Although multiple independent cohorts were incorporated for both training and validation, heterogeneity may still exist across datasets in terms of sample sources, experimental platforms, case composition, and completeness of clinical annotations. Secondly, the identification of GADD45A, HP, MAPK14, PGD, RETN, and S100A9 in this study was largely derived from bioinformatics-based screening and retrospective validation. Although the validation cohort did not participate in feature screening or model parameter tuning, the elevated area under the curve must still be interpreted with caution. Factors such as sample source, case composition, and batch processing methods in the public retrospective dataset may influence the model’s performance estimation. Therefore, the model’s generalization ability requires validation in a prospective, multicenter pediatric sepsis cohort in future studies. In addition, due to the limited availability of clinical variables in public databases, this study did not fully evaluate the combined predictive value of these candidate genes with conventional inflammatory markers, disease severity indices, or prognostic outcomes. This study has not yet incorporated the published adult sepsis biomarker signatures into the same pediatric validation cohort for the purpose of model reconstruction and direct performance comparison. Consequently, the current findings primarily underscore the diagnostic potential of oxidative stress-related genes in the pediatric sepsis cohort. Given this gap, the applicability of adult biomarker signatures to pediatric sepsis requires systematic evaluation in future studies, which should adhere to standardized preprocessing, feature definition, and modeling conditions. Furthermore, this study has not yet integrated the pediatric sepsis biomarker signatures identified in recent RNA-seq studies, including RORA, GPR183, and their associated high-confidence biomarker sets, into a cohesive preprocessing and modeling framework. Therefore, the comparative predictive performance of various pediatric sepsis biomarker signatures needs to be further evaluated under standardized training, validation, and evaluation conditions. Furthermore, the results obtained from immune cell deconvolution analysis, ceRNA network construction, and molecular docking primarily suggest potential biological associations and regulatory mechanisms. The analysis of immune cell composition in this study relied on transcriptome data from peripheral whole blood and the CIBERSORT deconvolution algorithm; however, this approach did not accurately reflect immune cell infiltration at the tissue level. Since alternative algorithms, including xCell, MCP-counter, and EPIC, were not employed for cross-validation, the findings should be regarded as exploratory estimates of the proportions of immune cells in peripheral blood. Future research must validate these results by using multiple deconvolution methods and independent samples. Computational screening and molecular docking provide insights into potential drug candidates; however, they do not directly demonstrate the therapeutic efficacy of these compounds in pediatric sepsis. The actual binding affinity and biological effects of the candidate compounds interacting with hub gene-encoded proteins require further validation through *in vitro* experiments, animal models, and clinical studies. Future studies should incorporate larger pediatric sepsis cohorts, longitudinal clinical data, and experimental validation to systematically evaluate the clinical utility of these oxidative stress–related candidate genes in early diagnosis, risk stratification, and the development of targeted therapeutic strategies.

## 6 Conclusion

Overall, bioinformatics analysis deepens our comprehension about sepsis pathophysiology as well as contribute to progress in more effective therapeutic interventions. Future laboratory-based experiments should confirm these findings to enable their immediate translation into clinical practice. The present findings are primarily based on publicly available transcriptomic datasets and retrospective cohort analyses. To establish robust diagnostic performance and enhance clinical translational relevance, further validation using prospective, multicenter clinical samples, together with experimental verification, is required.

## Data Availability

The complete analysis script has been uploaded to GitHub. https://github.com/Yumeng172/code.git.

## Supplementary material

SUPPLEMENTARY TABLE 1 Information on five transcriptome databases.

SUPPLEMENTARY TABLE 2 Six machine learning algorithms(Bayesian analysis, bagged trees, LASSO, RF, LVQ, and Boruta) for identifying candidate genes.

SUPPLEMENTARY TABLE 3 Screening results of candidate compounds.

SUPPLEMENTARY FIGURES S1-S2 Box plots describe data(GSE26440 and GSE26378) before and after batch effects.

SUPPLEMENTARY FIGURES S3-S4 Principal component analysis (PCA) plots describe data(GSE13904, GSE8121 and GSE9692) before and after batch effects.

SUPPLEMENTARY FIGURES S5-S6 Box plots describe data(GSE13904, GSE8121 and GSE9692) before and after batch effects.

SUPPLEMENTARY FIGURE S7 Weighted gene co-expression network analysis (WGCNA) of key module genes. **(A)** Hierarchical clustering of training queue samples. **(B)** Dynamic tree cut method for merging similar modular genes. **(C)** Scatter plot between turquoise module genes and disease trait.

SUPPLEMENTARY FIGURE S8 The ROC curve of S100A9, HP, PGD, MAPK14, GADD45A and RETN in the validation cohort.

SUPPLEMENTARY FIGURE S9 The Flowchart of main steps in molecular docking.
