# Supplementary material for: Identification and validation of biomarkers related to oxidative stress in pediatric sepsis utilizing WGCNA and multiple machine learning algorithms
Source: Front Bioinform. 2026 Oct 12;6:1745381. doi: 10.3389/fbinf.2026.1745381 (PMC13696922; doi:10.3389/fbinf.2026.1745381)
Supplement: Supplementary file 1 [file Table1.docx]

**The information of each dataset can be organized into the following format:**

|  | GSE26440 | GSE26378 | GSE13904 | GSE8121 | GSE9692 |
| --- | --- | --- | --- | --- | --- |
| **Platform** | GPL570 | GPL570 | GPL570 | GPL570 | GPL570 |
| **Species** | Homo sapiens | Homo sapiens | Homo sapiens | Homo sapiens | Homo sapiens |
| **Sample** | Peripheral blood | Peripheral blood | Peripheral blood | Peripheral blood | Peripheral blood |
| **Sepsis samples** | 98 | 82 | 67 | 30 | 30 |
| **Control samples** | 32 | 21 | 18 | 15 | 15 |
