# Supplementary material for: Identification and validation of biomarkers related to oxidative stress in pediatric sepsis utilizing WGCNA and multiple machine learning algorithms
Source: Front Bioinform. 2026 Oct 12;6:1745381. doi: 10.3389/fbinf.2026.1745381 (PMC13696922; doi:10.3389/fbinf.2026.1745381)
Supplement: Supplementary file 2 [file Table2.docx]

| Algorithm | Quantitative metric | Filtering criterion | Selected genes |
| --- | --- | --- | --- |
| LASSO | Non-zero coefficient/screening frequency | Lambda.min/1000 | ALDH1A1,DDAH2,GADD45A, GCLM, HP, MAPK14, MGST1, PGD, RETN, S100A9 |
| Boruta | Comparison between Z-score and shadow features |  | HP, RETN, LTF, MMP9, GADD45A, ARG1, CYP1B1, ADM, FKBP5, KCNE1, MAPK14, TSPO, TXN, ITGAM, F5, S100A9, MAP2K6, TIMP1, TLR2, CTSD, MGST1, GCLM, DDAH2, IL1R1, POR, IL1RN, ALOX5, MSRB1, PGD, PRKCD, ALDH1A1 |
| LVQ | variable importance | >0.5 | HP, RETN, LTF, MMP9, GADD45A, ARG1, CYP1B1, ADM, FKBP5, KCNE1, MAPK14, TSPO, TXN, ITGAM, F5, S100A9, MAP2K6, TIMP1, TLR2, CTSD, MGST1, GCLM, DDAH2, IL1R1, POR, IL1RN, ALOX5, MSRB1, PGD, PRKCD, ALDH1A1 |
| Bagged trees | Cross-validation performance | Optimal feature subset | RETN,HP,ARG1,S100A9,GADD45A, DDAH2, PGD, TSPO, LTF, KCNE1, CYP1B1, MAPK14, MMP9, MGST1, IL1RN, GCLM |
| RF | Cross-validation performance | Optimal feature subset | RETN, HP, ARG1, S100A9, DDAH2, GADD45A, KCNE1, PGD, LTF, MAPK14, CYP1B1, MGST1, IL1RN, MAP2K6, GCLM, TSPO, F5, ADM, ALDH1A1 |
| Bayesian | Cross-validation performance | Optimal feature subset | HP, RETN, DDAH2, KCNE1, TSPO, PGD, GADD45A, S100A9, ARG1, MAPK14 |
