# Supplementary material for: Identification and validation of biomarkers related to oxidative stress in pediatric sepsis utilizing WGCNA and multiple machine learning algorithms
Source: Front Bioinform. 2026 Oct 12;6:1745381. doi: 10.3389/fbinf.2026.1745381 (PMC13696922; doi:10.3389/fbinf.2026.1745381)
Supplement: Supplementary file 3 [file Table3.docx]

| Pubchem CID | Compounds | Targets | Affinity  (kcal/mol) | PDB ID | Method | Uniprot ID |
| --- | --- | --- | --- | --- | --- | --- |
| 54671203 | Doxycycline | MAPK14 | -8.4 | 4KIN | X-RAY DIFFRACTION | Q16539 |
| 11539025 | Ralimetinib mesylate | MAPK14 | -8.1 | 3LFF | X-RAY DIFFRACTION | Q16539 |
| 54682876 | Tasquinimod | S100A9 | -7.0 | 4GGF | X-RAY DIFFRACTION | P06702 |
| 4763 | Phenobarbital | PGD | -6.4 | 2KJV | X-RAY DIFFRACTION | P52209 |
